# Expert consensus on the prevention and treatment of enamel demineralization in orthodontic treatment

**DOI:** 10.1038/s41368-024-00335-7

**Published:** 2025-03-01

**Authors:** Lunguo Xia, Chenchen Zhou, Peng Mei, Zuolin Jin, Hong He, Lin Wang, Yuxing Bai, Lili Chen, Weiran Li, Jun Wang, Min Hu, Jinlin Song, Yang Cao, Yuehua Liu, Benxiang Hou, Xi Wei, Lina Niu, Haixia Lu, Wensheng Ma, Peijun Wang, Guirong Zhang, Jie Guo, Zhihua Li, Haiyan Lu, Liling Ren, Linyu Xu, Xiuping Wu, Yanqin Lu, Jiangtian Hu, Lin Yue, Xu Zhang, Bing Fang

**Affiliations:** 1 Department of Orthodontics, Shanghai Ninth People’s Hospital, Shanghai Jiao Tong University School of Medicine, College of Stomatology, Shanghai Jiao Tong University, National Center for Stomatology, National Clinical Research Center for Oral Diseases, Shanghai Key Laboratory of Stomatology, Shanghai Research Institute of Stomatology, Shanghai, China; 2 State Key Laboratory of Oral Diseases & National Clinical Research Center for Oral Diseases & Department of Pediatric Dentistry, West China Hospital of Stomatology, Sichuan University, Chengdu, China; 3 State Key Laboratory of Military Stomatology, National Clinical Research Center for Oral Diseases, Shaanxi Clinical Research Center for Oral Diseases, Department of Orthodontics, School of Stomatology, Air Force Medical University, Xi’an, China; 4 Department of Orthodontics, Hubei-MOST KLOS and KLOBM, School & Hospital of Stomatology, Wuhan University, Wuhan, China; 5 Department of Orthodontics, Affiliated Stomatological Hospital of Nanjing Medical University, Jiangsu Province Key Laboratory of Oral Diseases, Nanjing, China; 6 Department of Orthodontics, Capital Medical University School of Stomatology, Beijing, China; 7 Department of Stomatology, Union Hospital, Tongji Medical College, Huazhong University of Science and Technology, School of Stomatology, Tongji Medical College, Huazhong University of Science and Technology, Hubei Province Key Laboratory of Oral and Maxillofacial Development and Regeneration, Wuhan, China; 8 Department of Orthodontics, Peking University School and Hospital of Stomatology, National Center of Stomatology, National Clinical Research Center for Oral Diseases, National Engineering Research Center of Oral Biomaterials and Digital Medical Devices, Beijing Key Laboratory of Digital Stomatology, Beijing, China; 9 State Key Laboratory of Oral Diseases & National Clinical Research Center for Oral Diseases & Department of Orthodontics, West China Hospital of Stomatology, Sichuan University, Chengdu, China; 10 Department of Orthodontics, Hospital of Stomatology, Jilin University, Changchun, China; 11 Department of Orthodontics, Stomatological Hospital of Chongqing Medical University, Chongqing Key Laboratory of Oral Diseases and Biomedical Sciences, Chongqing Municipal Key Laboratory of Oral Biomedical Engineering of Higher Education, Chongqing, China; 12 Department of Orthodontics, Hospital of Stomatology, Guanghua School of Stomatology, Sun Yat-sen University, Guangdong Provincial Key Laboratory of Stomatology, Guangzhou, China; 13 Department of Orthodontics, Shanghai Stomatological Hospital, Shanghai Key Laboratory of Craniomaxillofacial Development and Diseases, Fudan University, Shanghai, China; 14 Center for Microscope Enhanced Dentistry, Capital Medical University School of Stomatology, Beijing, China; 15 Department of Operative Dentistry and Endodontics, Hospital of Stomatology, Guanghua School of Stomatology, Sun Yat-sen University, Guangdong Provincial Key Laboratory of Stomatology, Guangzhou, China; 16 Department of Prosthodontics, School of Stomatology, Air Force Medical University, State Key Laboratory of Oral & Maxillofacial Reconstruction and Regeneration, National Clinical Research Center for Oral Diseases, Shaanxi Key Laboratory of Stomatology, Xi’an, China; 17 Department of Preventive Dentistry, Shanghai Ninth People’s Hospital, Shanghai Jiao Tong University School of Medicine, College of Stomatology, Shanghai Jiao Tong University, National Center of Stomatology, National Clinical Research Center for Oral Diseases, Shanghai Key Laboratory of Stomatology, Shanghai Research Institute of Stomatology, Shanghai, China; 18 Tianjin Stomatological Hospital, School of Medicine, Nankai University, Tianjin Key Laboratory of Oral and Maxillofacial Function Reconstruction, Tianjin, China; 19 Department of Orthodontics, School of Stomatology, Harbin Medical University, the Second Affiliated Hospital of Harbin Medical University, Harbin, China; 20 Department of Orthodontics, Shenyang Stomatological Hospital, Shenyang, China; 21 Department of Orthodontics, School and Hospital of Stomatology, Shandong University, Jinan, China; 22 Department of Orthodontics, Affiliated Stomatological Hospital of Nanchang University, Jiangxi Provincial Key Laboratory of Oral Biomedicine, Nanchang, China; 23 Department of Orthodontics, School and Hospital of Stomatology, Hebei Medical University, Hebei Provincial Key Laboratory of Stomatology, Hebei Provincial Clinical Research Center for Oral Diseases, Shijiazhuang, China; 24 School of Stomatology, Lanzhou University, Lanzhou, China; 25 Department of Orthodontics, Fujian Key Laboratory of Oral Diseases, Stomatological Key Lab of Fujian College and University, School and Hospital of Stomatology, Fujian Medical University, Fuzhou, China; 26 Department of Orthodontics, School and Hospital of Stomatology, Shanxi Medical University, Shanxi Province Key Laboratory of Oral Diseases Prevention and New Materials, Taiyuan, China; 27 Department of Orthodontics, Xiangya Stomatology Hospital, Central South University, Changsha, China; 28 Department of Orthodontics, Affiliated Stomatological Hospital of Kunming Medical University, Kunming, China; 29 Department of Cariology and Endodontology, Peking University School and Hospital of Stomatology, National Center for Stomatology, National Clinical Research Center for Oral Diseases, National Engineering Research Center of Oral Biomaterials and Digital Medical Devices, Beijing Key Laboratory of Digital Stomatology, Beijing, China; 30 School and Hospital of Stomatology, Institute of Stomatology, Tianjin Medical University, Tianjin Key Laboratory of Oral Soft and Hard Tissues Restoration and Regeneration, Tianjin, China

**Keywords:** Dentistry, Risk factors

## Abstract

Enamel demineralization, the formation of white spot lesions, is a common issue in clinical orthodontic treatment. The appearance of white spot lesions not only affects the texture and health of dental hard tissues but also impacts the health and aesthetics of teeth after orthodontic treatment. The prevention, diagnosis, and treatment of white spot lesions that occur throughout the orthodontic treatment process involve multiple dental specialties. This expert consensus will focus on providing guiding opinions on the management and prevention of white spot lesions during orthodontic treatment, advocating for proactive prevention, early detection, timely treatment, scientific follow-up, and multidisciplinary management of white spot lesions throughout the orthodontic process, thereby maintaining the dental health of patients during orthodontic treatment.

## Introduction

The treatment course for correcting dental and maxillofacial deformities usually lasts 2 to 3 years. If treatment is started after the deciduous dentition period, it may extend even longer, possibly up to 7 to 10 years.^1,2^ During this period, poor maintenance of oral hygiene and a lack of health education and management can lead to a demineralization-remineralization imbalance of the hard dental tissues around orthodontic appliances and gingival margins. Mineral loss beneath the relatively intact enamel surface causes an increase in surface porosity, resulting in decreased translucency and loss of gloss, resulting in white spot lesions (WSL) on the enamel surface.^3^ WSL is softer in texture than adjacent healthy enamel, appearing chalky white when dry. Some patients may experience decreased satisfaction due to the aesthetic effect of white spots on the enamel surface after orthodontic appliances removal. As WSL progresses, surface enamel collapses and caries form. In severe cases, pulp tissue may invade, causing pulpitis, which requires root canal treatment or even extraction of the affected tooth.^4–7^ Therefore, the prevention, early diagnosis, and treatment of WSL during orthodontic treatment are highly important for maintaining oral health and enhancing aesthetics and patient satisfaction after orthodontic treatment.^8^

The prevalence of WSL ranges from 23.4% to 75.6%, depending on the detection methods and research purposes. Patients wearing clear aligners have a lower incidence of WSL than those receiving treatment with fixed appliances.^9,10^ The severity of WSL in males was greater than that in females, although the incidence of WSL was not significantly different according to sex. In orthodontic cases, WSL affects 23.4% of anterior teeth, with a greater incidence of maxillary anterior teeth than mandibular anterior teeth. Additionally, the WSL in patients wearing clear aligners appear larger but shallower, while those in fixed appliance patients tend to be smaller but deeper.^11^ Risk factors for WSL include dental fluorosis, orthodontic treatment for more than 36 months, poor oral hygiene before treatment, deterioration of oral hygiene during orthodontic treatment, and preexisting WSL. The most significant risk factor is the presence of preexisting WSL, followed by deterioration of oral hygiene during treatment and poor oral hygiene before treatment.^12–14^ Studies have shown that fixed orthodontic appliances are difficult to clean in the oral cavity, leading to increased plaque accumulation, which lowers the pH around them and increases the risk of caries.^15,16^ Studies also report that the composition of the dental plaque biofilm microflora changes after wearing orthodontic appliances, with significantly elevated levels of acidogenic bacteria, including mutans streptococci. These bacteria, when provided with sufficient carbohydrates, produce acidic byproducts, further lowering the plaque pH below the remineralization threshold and disrupting the mineralization-remineralization balance, leading to demineralization of dental hard tissues and ultimately causing WSL over time.^17,18^

The main clinical treatment methods for WSL involve reducing enamel demineralization, promoting remineralization, and aesthetically restoring demineralized enamel. Fluoride is a commonly used preventive agent for WSL. It forms fluoroapatite and fluorohydroxyapatite by binding with calcium and phosphate in the enamel. As shown in Table 1, these substances have higher solubility products than hydroxyapatite, making them more resistant to acid dissolution, thus enhancing the acid resistance of teeth to reduce enamel demineralization. However, fluoride application must be within the nationally regulated safe dosage limits, as excessive use can cause fluorosis. Remineralizing agents such as casein phosphopeptide-amorphous calcium phosphate (CPP-ACP) work by promoting the repair of microlesions on the enamel surface, allowing calcium and phosphate ions to redeposit on the enamel, restoring its structure, and inhibiting WSL progression. However, their efficacy in severe cases is limited. Aesthetic restoration of teeth affected by WSL, which involves removing a certain amount of superficial enamel, is considered the best approach for restoring dental configuration.^19,20^ Various limitations exist in the treatment of severe WSL patients, emphasizing the importance of timely detection of WSL during orthodontic treatment and intervention with appropriate treatment methods. This article provides guidance on the management and treatment of WSL in orthodontic cases.

**Table 1:** Common calcium phosphate compounds and corresponding solubility products

Calcium Phosphate Systems	Abbreviation	Chemical structure	Ksp
Calcium Hydrogen Phosphate	DCPD	CaHPO_4_·2H_2_O	6.6
β-Tricalcium phosphate	*β*-TCP	*β*-Ca_3_(PO_4_)_2_	29.5
Octacalcium phosphate	OCP	Ca_8_H_2_(PO_4_)_6_·5H_2_O	98.6
Hydroxyapatite	HA	Ca_10_(PO_4_)_6_(OH)_2_	117.2
Fluorapatite	FA	Ca_10_(PO_4_)_6_(F)_2_	120.3
Amorphous calcium phosphate	ACP	Ca_3_(PO_4_)_1.87_(HPO_4_)_0.2_	24.8

**Fig. 1:**
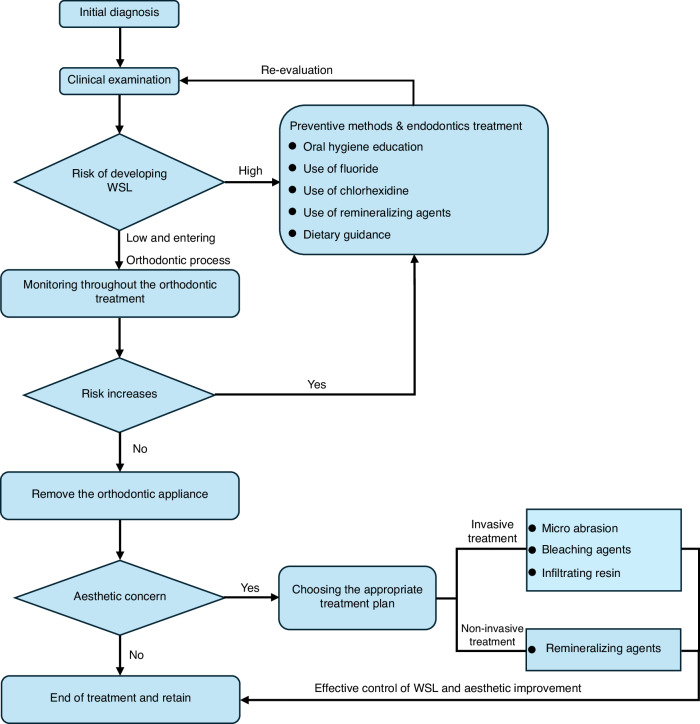
Recommended clinical procedure flowchart for WSL treatment

The recommended clinical procedure is shown in Fig. 1. At the initial visit, the patient’s risk of dental demineralization should be assessed through clinical examination and medical history inquiry. If evaluated as high risk, preventive methods should be implemented, and a referral to endodontics is recommended. Orthodontic treatment should only commence if the patient is assessed as low-risk. During orthodontic treatment, the risk of dental demineralization should be evaluated at each follow-up visit, with timely preventive methods and referrals to endodontics as needed until the risk is reduced. After the removal of orthodontic appliances, appropriate invasive or non-invasive treatment measures should be selected for teeth with aesthetic concerns to achieve satisfactory outcomes for the patient. The following sections will detail the key points of each stage.

## Diagnosis of enamel demineralization

**Fig. 2:**
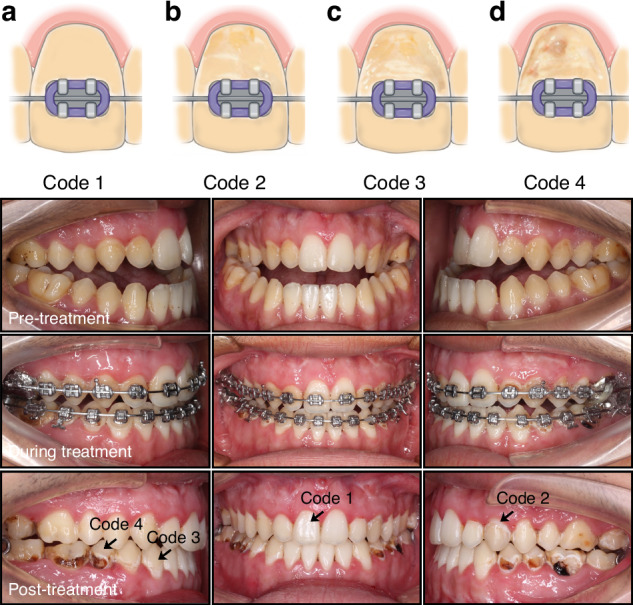
Diagrammatic depiction of the method used for lesion scoring and the intraoral photographs of the appearance of WSLs in the progress of orthodontic treatment. **a** (code 1): enamel with smooth tooth surface; **b** (code 2): enamel surface with linear lesions; **c** (code 3): enamel surface with striped lesions and **d** (code 4): enamel surface with cavitation

Gorelick et al. proposed a scoring system based on the severity of WSL, and Fig. 2 shows the scoring criteria and the intraoral photographs of the appearance of WSLs in the progress of orthodontic treatment.^12^

There are various kinds of clinical examination methods for WSL. The most used methods include visual examination and digital photo evaluation. In recent years, technologies such as fluorescence, electrical resistance testing, light-conducting fiber transillumination, and near-infrared transillumination have gradually been applied in the clinical diagnosis of WSL.^21,22^

### Oral examination

The most commonly used method for diagnosing WSL is visual examination.^23^ By combining visual examination with probing, it can be determined whether the WSL is in a stable period. A rough and chalky enamel surface indicates active demineralization, while a smooth and glossy enamel surface indicates a balance between demineralization and remineralization, with no further development.^24^ The refractive index of healthy enamel for light is 1.62, indicating semitransparency. However, the refractive index of demineralized enamel increases due to increased porosity, resulting in a chalky appearance. When the surface of demineralized enamel is moist, water fills the tiny gaps. As the refractive index of water is close to that of healthy enamel (1.33), the chalky appearance of demineralized enamel is difficult to observe. Dry demineralized enamel, on the other hand, filled with air with a refractive index of 1.0 in its tiny gaps, exhibits a noticeable chalky appearance.^25^ Therefore, for accurate visual examination, the tooth surface needs to be cleaned, dried for at least 5 seconds, and examined with the assistance of a mouth mirror and bright light. Visual examination has the advantages of simplicity and cost-effectiveness, without the need for additional equipment. However, this method has disadvantages such as subjectivity in examination, low repeatability, and difficulty in accurately diagnosing advanced lesions.^26^

### Digital photo evaluation

A simple visual examination cannot provide patient records. Oral photographs taken with a digital camera can store data on dental lesions for consultation among orthodontists, prosthodontists, endodontists, and preventive dentistry departments, facilitating remote discussions on shared examination results. Before taking photographs, it is necessary to carefully clean the tooth surface to remove plaque, dry the tooth surface and maintain an appropriate distance between the camera and the target tooth. It is recommended to use horizontal dual flash or ring flash and a camera with interchangeable macro lenses. After obtaining high-definition images, the tooth WSL value was calculated by defining regions of interest (ROIs) on the tooth surface and analyzing the grayscale values.^27^ Digital photos can store patient data for a long time with greater repeatability, but they have drawbacks such as technical sensitivity and a high cost of equipment.^28^

### Fluorescence technology

When teeth are exposed to light of specific wavelengths, fluorescence is produced. The fluorescence intensity varies among enamel, dentin, and cementum.^29^ Because dentin contains more organic material, its intrinsic fluorescence intensity is greater than that of enamel. After demineralization, the intrinsic fluorescence of the enamel decreases, and these optical changes are directly related to the mineral content of the enamel. Therefore, utilizing the spontaneous fluorescence characteristics of teeth for diagnosing demineralization has several applications. Quantitative light-induced fluorescence (QLF) is a technique that utilizes this fluorescence principle. The tooth surface was irradiated with near-ultraviolet light, and the generated fluorescence image was analyzed using specific software to display the size and density of demineralized enamel lesions.^30^ Some laser devices emit red light at wavelengths of 638-655 nm to diagnose demineralization of tooth tissue by inducing infrared fluorescence after irradiation. The fluorescence intensity increases with the severity of demineralization, but its precision is insufficient to measure tiny variations in mineral content.^31^ To enhance diagnostic sensitivity, a dye-enhanced laser fluorescence (DELF) technique has been developed. It involves staining demineralized tissues with a fluorescence dye that penetrates and enhances the fluorescence generated by the laser by combining with a fluorescence dye spectrum close to the wavelength of the DIAGNOdent laser.^32,33^ However, fluorescence technology also has significant drawbacks. Factors such as tooth staining, prosthesis, and other restorative materials can affect the fluorescence signal, leading to false-positive or false-negative results.^34^ Research has shown that the effectiveness of the fluorescence detection device DIAGNOdent Pen is comparable to that of conventional visual inspection. In recent years, various devices based on fluorescence technology have been developed, such as DIAGNOdent (KaVo, Germany), MidWest (DENTSPLY, USA), VistaProof (Durr Dental, Germany) and others.^35^ Although various fluorescence methods for caries detection devices are currently used in clinical practice as auxiliary diagnostic tools for caries detection, they cannot yet be considered the gold standard for detecting dental caries.^36^

### Fiber-optic transillumination—digital imaging fiber-optic transillumination (FOTI-DIFOTI)

The light transmission coefficient of demineralized dental tissue differs from that of healthy dental tissue. Demineralization disrupts the dense hydroxyapatite in dental tissue, causing light to scatter as it passes through the highly porous demineralized tissue, resulting in optical distortion. Since the light transmission coefficient of intact enamel is greater than that of carious lesions, shadows can be seen when examining demineralized dental tissue with a fiber optic device.^37^ By evaluating the shadow intensity formed by the device’s light, demineralized dental tissue can be distinguished. Near-infrared light transillumination (NILT) uses longer wavelengths of invisible near-infrared light to reduce light scattering within dental tissue, allowing better penetration of dental tissue.^38,39^ As a result, this method can provide high contrast between healthy tissue and demineralized lesion tissue. A recent study showed that this diagnostic method can more accurately detect early demineralization of dental enamel and dentin hidden in dental tissue than other methods.^40^ The principle of light transmission through optical fibers is the scattering and absorption of light on the surface of enamel and dentin, stains, pigments, etc., on the surface of teeth, which may cause false positives. Fillings, prostheses, etc., can also cause the corresponding areas to be undetectable.^41^ In recent years, fiber optic transillumination technology has been widely used clinically to detect smooth surface caries, proximal caries, secondary caries, dental fluorosis, incomplete fracture, etc.^42^

### Electrical resistance measurements

Electrical resistance measurement devices for dental hard tissues use fixed-frequency AC to measure their resistance.^43^ The magnitude of the resistance depends on the porosity of the measured tooth area, the amount of liquid in the porous areas, the temperature, the flow of liquid, and the ion concentration in the porous areas. It has been reported that the accuracy of resistance measurements in demineralized dental tissues on sound surfaces is greater than that on occlusal surfaces.^44–46^ In the early stages of lesions, the sensitivity of electrical resistance measurement is superior to that of fiber optic transillumination, but its repeatability is poor, with some clinical limitations and less clinical application.

### Optical coherence tomography

In addition to the methods mentioned above, in recent years, swept-source optical coherence tomography (SS-OCT) has been increasingly utilized in dentistry. SS-OCT works by directing weak, coherent light onto the tooth surface. As demineralization of enamel occurs, organic components increase, leading to a decrease in the light scattering coefficient compared to surrounding healthy tissue. This reduces light intensity, presenting as a high-gray image compared to the surrounding healthy area, enabling the detection and analysis of early enamel caries.^47^ Studies have reported that SS-OCT can effectively assess the depth of WSL and has been applied in clinical practice.^48^

### Artificial intelligence (AI)

With the advancement of artificial intelligence technology, deep learning has made significant progress in dentistry. Convolutional neural networks (CNNs) based on deep learning have been widely used in cervical vertebral maturation staging, automatic landmarking of lateral cephalograms, and caries diagnosis due to their advantages in processing large images. Research has shown that using CNNs for segmenting digital dental surface photos and digital fiberoptic transillumination images can achieve an automatic caries detection accuracy of up to 95%.^49^

AI models have shown excellent diagnostic performance in caries detection and may become an important auxiliary tool in clinical practice. Future research needs to rely on comparable, large, and clinically significant datasets.

### Other methods

In recent years, high-frequency ultrasound (HFUS) for measuring enamel demineralization and photothermal radiometry (PTR) have also been explored for the clinical detection of enamel demineralization. However, further research is needed to develop these detection methods.^50,51^

The International Caries Detection and Assessment System (ICDAS) 2004 consensus workshop concluded that visual examination and probing remain the standards for caries diagnosis.^23^ Currently, there are a variety of methods used for demineralization detection, serving as auxiliary tools for clinical decision-making, enhancing diagnostic accuracy, and monitoring disease progression.^52^

### Clinical recommendations for the diagnosis of enamel demineralization

- The preferred method for examining the demineralization of tooth surfaces is through a combination of visual and probing examination, which can be supplemented by using a digital camera and a macro lens to record the demineralized tooth surfaces. It is important to ensure sufficient light but avoid overexposure when taking photos with a digital camera to prevent false-negatives.
- During the examination of tooth surfaces, it is important to thoroughly clean and dry the surfaces and observe them under bright light to detect any changes in the appearance of white chalkiness.
- A probe was used to examine the roughness of the tooth surfaces when conducting the examination and to assess whether demineralization was in an active stage.
- For quantitative analysis of white chalky changes on tooth surfaces, supplementary methods such as fluorescence technology, fiber optic transillumination, and resistance testing should be used. The use of artificial intelligence for interpreting tooth demineralization has a promising application in assisting chair-side examinations.

## Risk assessment of enamel demineralization before orthodontic treatment

The orthodontic appliances used during treatment may increase the area of plaque attachment, making it difficult to clean the appliance and the surrounding tooth surfaces. The irregular surfaces of brackets, archwires, and bands may limit the movement of oral muscles and the natural self-cleaning action of salivary glands, which makes plaque formation easier.^53^ WSL are early signs of dental caries, with contributing factors including the host, bacteria, food, and time. Reducing plaque formation and decreasing the consumption of cariogenic foods are primary strategies for minimizing WSL development. WSL is a significant factor that compromises oral health and aesthetics throughout orthodontic treatment. Therefore, risk assessment and oral health education before orthodontic treatment play a vital role in reducing the occurrence of WSL.^54,55^

**Fig. 3:**
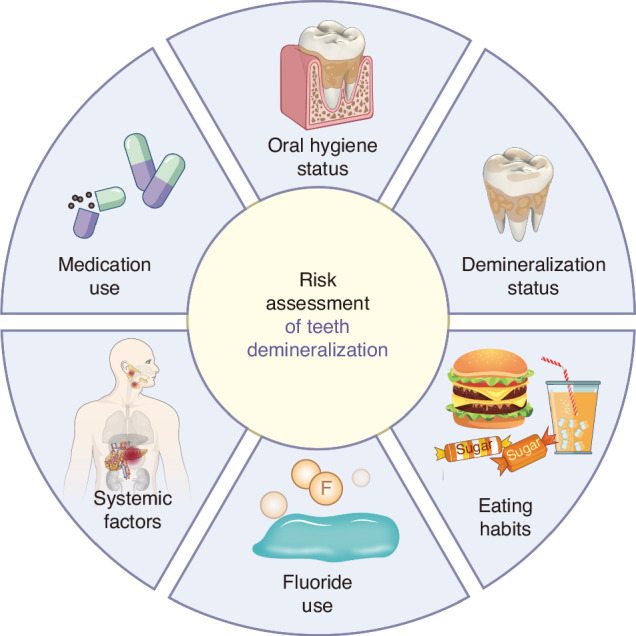
Summary diagram of demineralization risk assessment

Risk assessment for WSL before orthodontic treatment includes the following aspects. Additionally, Fig. 3 briefly illustrates the primary risks associated with dental demineralization.

### Oral hygiene status

The use of fixed orthodontic appliances during orthodontic treatment increases irregularities on the tooth surface, providing conditions for plaque attachment and retention, thus increasing the difficulty of oral cleaning. Poor oral hygiene habits and inadequate tooth brushing can lead to plaque accumulation on the tooth surface, potentially causing enamel demineralization, resulting in WSL.

### Demineralization status

To determine a patient’s level of demineralization risk, a comprehensive medical history should be taken, including a series of questions related to known risk factors for increased caries susceptibility or protective factors. This involves assessing the patient’s medical history and relevant social history (e.g., place of birth and upbringing, current residence, educational level, and occupation). All this information is crucial for evaluating demineralization risk at an individual level.^56^ By assessing the patient’s past caries experience, one can reflect their susceptibility to dental demineralization.

### Eating habits

The dietary structure, especially the frequency and quantity of fermentable carbohydrate intake, and the impact of diet on oral pH were evaluated.

### Fluoride use

The use of fluoride-containing toothpaste, mouthwash, gels, etc., and whether the patient received professional fluoride varnish or other fluoride-releasing material treatments were also assessed. Fluoride is an effective anticaries agent that promotes the remineralization of dental enamel, inhibits plaque metabolism, and increases enamel acid resistance. The regular use of fluoride-containing toothpaste, mouthwash, or gels or the use of professional fluoride varnish or other fluoride-releasing materials during orthodontic treatment can reduce the occurrence of WSL.

### Systemic factors

The presence of systemic diseases, long-term medication use, or other factors that affect saliva secretion and oral microbiota balance were assessed. Some systemic diseases, medications, or other factors may also affect the risk of WSL during orthodontic treatment, such as decreased saliva secretion, weakened immunity, diabetes, and Sjogren’s syndrome.

## Preventive methods for enamel demineralization in orthodontic treatment

To reduce the occurrence of WSL before orthodontic treatment, oral health education needs to be part of the treatment plan, including the following aspects:

### Oral hygiene instruction and health education

Regular professional endodontic examination helps to detect early white chalky spots or caries on the tooth surface and to see the specialist in time.^57^ Regular professional periodontal therapy should be carried out to remove plaque and calculus, prevent decreased pH on the tooth surface, and prevent damage to the enamel. For orthodontic patients with periodontal disease, regular periodontal examination and treatment should also be conducted to control periodontal inflammation and biofilm formation.

### Oral health care

The teeth were brushed promptly after each meal, at least 3 times a day, with each tooth lasting no less than 3 min. Special orthodontic dental floss can be used to clean gaps through brackets and archwires. If it is necessary to clean the interproximal gaps between teeth, it must be performed under the f the doctor’s teaching. The food residue on the tooth surface and around the orthodontic appliances was cleaned with a gentle up-and-down brushing motion to reduce plaque accumulation. In addition, disclosing agents can be used after brushing to more clearly show areas that still need cleaning, increasing the interest in brushing teeth of patients, especially children, and further improving the effect of brushing teeth.

### Use of fluoride

Fluoride reduces demineralization of dental tissues via three different mechanisms.^58–60^ The first mechanism is that the presence of fluoride increases the formation and accumulation of fluorapatite. Fluorapatite is formed by the combination of calcium ions and phosphate ions in saliva and has lower solubility than hydroxyapatite, increasing the acid resistance of enamel.^61,62^ The second mechanism is promoting the direct remineralization of fluorapatite crystals on the surface of dental tissues undergoing demineralization. The third mechanism relies on the antibacterial activity of fluoride ions, and low concentrations of fluoride can inhibit the production of glucosyltransferases. The main role of glucosyltransferases is to increase bacterial adhesion and provide glucose for extracellular polysaccharide formation. Local fluoride application at high concentrations (12000 ppm) has a direct toxic effect on oral microbiota, including *Streptococcus mutans*.^63^

**Fig. 4:**
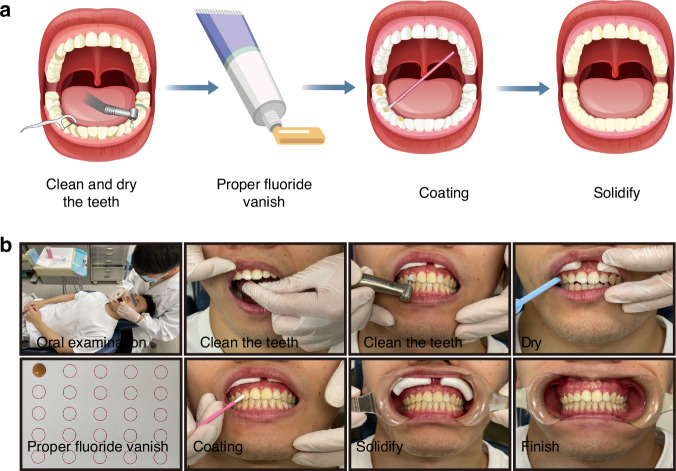
Fluoride application procedure. **a**: Schematic diagram of the fluoride varnish application process on tooth surfaces. **b**: Intraoral photographs of the fluoride application process

Fluoride can be classified as systemic or topical. Systemic application is effective for individuals at high risk of caries or in low-fluoride areas. According to the World Health Organization’s report, a daily intake of 1 mg of fluoride is beneficial to health. Systemic fluoride application methods include adding fluoride to drinking water, salt, and milk, as well as adding fluoride-containing tablets or drops to the diet. Topical applications, such as fluoride varnish, can extend the contact time with teeth and slowly release fluoride to prevent the rapid loss of fluoride after use^64,65^. The American Academy of Pediatric Dentistry recommends using fluoride varnish at least 5% (22,600 ppm) on primary teeth twice a year and 2-4 times a year on permanent teeth.^66,67^ As shown in Fig. 4, the process of applying topical fluoride is relatively simple. Briefly, the tooth surface is first cleaned and dried. Then, an appropriate amount of fluoride is applied to the tooth surface. It is important to note that no eating should occur within 2 to 4 h after the fluoride application, and brushing should be avoided that evening to ensure the effectiveness of the application.

### Dietary guidance

Forms of good eating habits include avoiding or reducing the consumption of sticky, hard, acidic, and high-sugar foods, such as candies and carbonated drinks, to prevent tooth erosion. If the individual has snacks between meals or drinks carbonated drinks or milk tea, it is advisable to brush their teeth or rinse their mouths promptly. The oral cavity was cleaned thoroughly before sleeping at night, ensuring that no food or drink residue remained to prevent the formation of dental plaque that can corrode teeth. Improving oral hygiene and reducing the intake of cariogenic foods are essential measures for patients to reduce biofilm attachment and decrease acid-producing bacteria (such as *Streptococcus mutans*) metabolism, effectively preventing the formation of WSL.^68^ Educating patients on healthy lifestyles is the best way to prevent oral health problems.

### Use of chlorhexidine

Chlorhexidine (CHX) can significantly inhibit the growth of *Streptococcus mutans*, accelerating its remineralization. CHX is available in varnishes, gels, and aqueous solutions. Studies have shown that protective varnish Cervitec F containing CHX can achieve persistent inhibition of *Streptococcus mutans* compared to gels and mouth rinses while also reducing the incidence of WSL.^69–71^ Although the safety of CHX has been confirmed, it can lead to side effects such as taste disturbance, oral mucosal staining, and contact dermatitis. For orthodontic treatments with longer durations, the localized application of CHX varnish on the tooth surface is safer.^72,73^

### Key points for preventing demineralization with various orthodontic appliances

Orthodontic treatments typically involve fixed appliances such as brackets on the labial or lingual side or clear aligners. Due to differences in the structure and placement of these appliances in the patient’s oral cavity, methods to prevent enamel demineralization vary. For fixed appliances bonded to the tooth surface, such as brackets, the presence of brackets and archwires hinders self-cleaning of the oral cavity and daily hygiene, requiring brushing and cleaning of food debris around the brackets after every meal to reduce plaque accumulation.^74^ Compared to fixed appliances, clear aligners are transparent, removable, thermoplastic orthodontic devices that cover a larger area of the tooth surface. Although clear aligners can be easily removed for cleaning, they need to be worn for 20 to 22 h daily. Failing to maintain good oral hygiene during wear could lead to severe enamel demineralization, presenting as large and shallow areas of demineralization on the teeth.^75^ Therefore, before wearing clear aligners, it is important to clean the tooth surface. After drinking sugary drinks while wearing them, rinsing the mouth promptly is necessary to prevent acidification that could lead to demineralization of tooth tissues.

### Clinical recommendations for preventing enamel demineralization in orthodontic treatment

- Maintaining good oral hygiene is the primary method for preventing enamel demineralization in orthodontic treatment. Oral hygiene instructions and health education are crucial.
- Emphasis should be placed on the correct and effective toothbrushing method, ensuring both the duration and frequency of brushing and reducing the intake of cariogenic foods.
- The use of fluoride toothpaste for daily dental care should be encouraged to enhance the acid resistance of enamel and reduce demineralization.
- After wearing orthodontic appliances, it is essential to clean the oral cavity and the area around the appliances for food residue after each meal to prevent the formation of an acidic environment leading to enamel demineralization.

## Management of enamel demineralization during orthodontic treatment

WSL on the tooth surface is a common complication in orthodontic patients with poor oral hygiene.^76^ Factors such as microleakage at the bracket-adhesive-bracket interface, prolonged acid etching during bonding, extended orthodontic treatment duration, and lack of oral health awareness in patients can all contribute to plaque accumulation, acid production by bacteria, decreased enamel pH, and subsequent enamel demineralization. Early detection of white spot lesions during orthodontic treatment and timely implementation of appropriate management strategies are crucial for safeguarding dental health.

### Assess demineralization risk throughout the process of orthodontic treatment

During orthodontic treatment, it is crucial to assess whether the patient’s risk of demineralization has increased during follow-up visits. This assessment includes evaluating oral hygiene status, such as plaque accumulation, calculus deposition, and food impaction. If the risk of demineralization is found to be elevated, preventive methods and endodontic treatment should be promptly employed to prevent the occurrence of dental demineralization. This includes the use of fluoride toothpaste, fluoride varnish, and mouth rinses containing fluoride around orthodontic appliances.^77^

### Remineralization and antibiofilm combined therapy

The remineralization agent casein phosphopeptide-amorphous calcium phosphate (CPP-ACP) stabilizes ACP by incorporating phosphorylated serine from casein, thus maintaining a state of supersaturation with Ca^2+^ and PO_4_^2-^ on the tooth surface and promoting the remineralization of hard dental tissues.^78^ CPP-ACP is a good choice for WSL remineralization, and when combined with fluoride, it enhances the remineralizing effects of WSL.^79,80^ Although the application of fluoride, CPP-ACP, and others has a positive effect on preventing WSL progression, these methods still lack aesthetic improvement,^81^ and further clinical evidence is needed to prove the effectiveness of CPP-ACP in promoting WSL remineralization.^82^ Therefore, materials capable of stabilizing and delivering ACP directly to the tooth surface, apart from fluoride, are indeed among the best choices for remineralizing hard dental tissues. With the ongoing research and translation of new biomimetic remineralization materials, the use of remineralizing agents is certainly a robust means for the prevention and treatment of WSL.

Based on the concept and technology of interrupting dental caries (IDC), in recent years, biomaterials targeting biofilms and remineralization have been continuously emerging. Poly(carboxylic acid) succinyl chitosan acrylamide (PCBAA)/ACP nanocomposite materials not only provide ions but also prevent rapid aggregation and spontaneous transformation of ions on the lesion surface, allowing calcium and phosphate ions to penetrate the gaps more effectively, accelerating internal crystal growth, and promoting the formation of dense remineralization layers.^83^ Phase transition bovine serum albumin-octopamine (PTB-OCT) is a universal anticaries coating that not only induces mineralization on the surface of hard dental tissues and resins but also exhibits acid resistance and antimicrobial properties, reducing primary caries and postfill microleakage.^84^ L-cysteine/graphiticyne/silver composite nanozymes (GDY/L-cys/Ag, GLA) inhibit dental plaque by producing reactive oxygen species, and GLA serves as a nucleation point, cross-linking with saliva rich in Ca^2+^, attracting PO_4_^3-^, promoting the formation of hydroxyapatite on enamel, and facilitating rapid remineralization[83]. the polymeric nature of invisible aligner materials, future basic research and clinical translation may involve developing polymeric materials with fluoride ion slow-release capabilities, antimicrobial properties, or surface modifications to resist biofilm formation.

### Laser therapy

Carbon dioxide lasers with wavelengths of 9.3, 9.9, 10.3, and 10.6 μm are considered the main types of lasers for inhibiting demineralization of dental tissues, as the absorption bands of phosphates, carbonates, and hydroxyl groups in enamel and dentin structures fall within the range of 9–11 μm. Laser absorption by enamel leads to physical and chemical changes, including organic matrix decomposition, reduced carbonate compounds, and fusion and recrystallization of hydroxyapatite crystals, resulting in increased acid resistance.^85–87^ Furthermore, research shows that low-energy lasers can reduce enamel demineralization by 90%.^88,89^ An Er: YAG laser with a wavelength of 2.94 μm is absorbed by water, hydroxyapatite, and collagen.^90–92^ The subablative energy of the Er: YAG laser induces chemical changes in dental tissues without causing morphological damage. Studies suggest that the combined use of lasers and fluoride can synergistically enhance the anti-demineralization ability of dental tissues, but clinical evidence is still lacking, with research remaining in the preclinical stages.^93–96^

### Ozone use

Ozone is a potent oxidizing agent that is destructive to various pathogens, displaying germicidal, antiviral, and antifungal activities by enhancing tissue metabolism through oxidation.^97,98^ Studies have shown that the use of ozone can reduce the counts of streptococci and candida in saliva.^99^ The safe concentration range for ozone is 0.05-5%, and ozone is generally applied in the form of gas, gels, or aqueous solutions in the oral cavity and has good biocompatibility. OzonyTron-OZ (Mymed, Germany) is an intraoral ozone gas disinfection device that kills pathogens within demineralized teeth by adjusting the silicone tray to adhere to the tooth surface and ozone gas flow, preventing further demineralization of the teeth.^100^ However, the clinical application of ozone for remineralization still requires more evidence and appropriate research.^101,102^

### Microabrasion of dental hard tissues

The main indication for microabrasion of dental hard tissues is intrinsic discoloration or texture changes caused byamelogenesis imperfecta or dental fluorosis.^103^ This technique uses slow-speed dental handpieces with gels containing acid and abrasives to remove discoloured enamel and stains the tooth surface.^104,105^ Microabrasion is a minimally invasive treatment that involves removing a certain amount of dental tissue. When removing the WSL from dental tissue, attention should be given to the thickness of the enamel in the cervical area.^106^ Research has shown that microabrasion improves the aesthetics of teeth with white spot lesions and demonstrates durability for at least 12 months. However, compared to microabrasion, resin infiltration has better aesthetic improvement effects after 12 months.^107,108^

### Use of bleaching agents

In vitro studies have shown that bleaching can improve the aesthetics of teeth with WSL. However, the bleaching process only enhances the appearance, disguising white spot lesions instead of treating them.^109^ Although bleaching WSL in vitro can reduce the differences in color between carious and unaffected areas, there is still no clear evidence for its clinical application.^110,111^

### Resin infiltration treatment

During WSL development, there is an increase in microporosity in the enamel. Low-viscosity light-curing resin infiltrates the microporous enamel area of WSL through capillary action, sealing the micropores and increasing the strength of the enamel, providing mechanical support to inhibit the progression of WSL.^112–116^ During WSL treatment, resin infiltration results in better aesthetic results than minor adjustments. The ability to camouflage WSL is mainly due to the refractive index of the infiltrated resin being close to that of hydroxyapatite crystals.^104,117,118^

In conclusion, different treatment options can be chosen based on the severity of the WSL that occurs during orthodontic treatment. For WSL with a Gorelick score of 3 or below, the localized use of fluoride combined with remineralizing agents may promote enamel remineralization.WSL with a score of 3 or above would require minimally invasive treatment.

## Clinical management and treatment of postorthodontic WSL

After orthodontic treatment is completed and the orthodontic appliance is removed, the health of the dental hard tissues and periodontal tissues needs to be reassessed. When necessary, consultation and treatment from disciplines such as dental pulp therapy, prosthodontics, periodontics, and preventive dentistry may be needed.

## Other considerations in orthodontics: management of enamel demineralization in early orthodontic treatment for children and adolescents

Due to the young age and long duration of orthodontic treatment in children and adolescents, awareness of maintaining good oral hygiene is often lacking. Since enamel mineralization in children and adolescents is incomplete, the risk of developing WSL during orthodontic treatment is greater. Uncontrolled progression of WSL can lead to dental caries and even pulp disease, affecting tooth development.^119^ Oral hygiene guidance for children and education for parents are necessary prior to orthodontic treatment for children and adolescents. If needed, fluoridated toothpaste should be used for brushing, along with disclosing agents to maintain good oral hygiene. Early pit and fissure sealants for molars should be applied. During each follow-up visit, monitoring of oral hygiene status, such as plaque, calculus, and gum health, should be conducted to prevent the causative factors of WSL. If WSL has already developed during orthodontic treatment, localized fluoride application, the use of remineralizing agents, or resin infiltration can be considered for treatment. Further oral hygiene education for children and parents is essential.^120^ If the WSL continues to progress, it may be necessary to replace the orthodontic appliance with one that is easier to clean or to temporarily remove the appliance until effective control of the WSL is achieved before continuing orthodontic treatment.

## Recommended clinical procedures for the prevention and treatment of WSL during the whole orthodontic treatment process

The management of WSL should start at the first visit before orthodontic treatment and become part of the treatment plan. Health education throughout the orthodontic process should aim to improve patients’ lifestyle for better oral health, emphasizing prevention over treatment. Treatment should initially involve conservative, noninvasive, and reversible methods. If these methods do not effectively resolve WSL, a more proactive approach may be necessary (Fig. 1).^121–124^

- Before orthodontic treatment, it is necessary to fully evaluate the risk factors for dental caries. Only when the risk factors are under control, the subsequent orthodontic treatment could proceed.
- During orthodontic treatment, it is essential to monitor the occurrence of WSL at each follow-up visit and intervene promptly. The primary focus should be on enhancing oral hygiene education, maintaining good oral health, and using fluoride and remineralizing agents locally to promote WSL remineralization.
- If the progression of WSL on the tooth surface during orthodontic treatment is uncontrollable, replace the orthodontic appliance with an easier-to-clean appliance or temporarily suspend orthodontic treatment until the WSL is effectively controlled.
- After orthodontic treatment, a multidisciplinary approach should be taken based on the severity of tooth demineralization after appliance removal.
- For orthodontic treatment in children and adolescents, oral hygiene education is necessary for guardians to ensure patient compliance with treatment and reduce the occurrence of WSL.

## The research prospects of WSL treatment

As the person in charge of orthodontic treatment, dentists providing orthodontic treatment have a responsibility to conduct in-depth research on the mechanism of WSL so that the technological means in the clinic and basic research in the laboratory complement each other, using a combination of measures to increase the prevention and treatment of WSL to new heights. In the field of basic research, it is possible to study biomimetic remineralization methods for rapid and efficient restoration of natural/synthetic enamel hard tissues and combine them with antibiofilm technologies to block WSL in the early stages before it develops into severe caries. In the future, orthodontists can closely collaborate and cooperate with oral prevention physicians, dental pulp physicians, and oral material researchers to develop a material that not only resists biofilms but also rapidly and effectively repairs demineralized enamel in a form closest to natural occurrence to reduce the risk of WSL during orthodontic procedures, ultimately aiming to improve the effectiveness of orthodontic treatment.

WSL involves not only a continuous loss of minerals in teeth but also a dynamic process of demineralization and remineralization. It is an early manifestation of caries. The progression of the disease depends on the balance between the pathological factors of demineralization (cariogenic bacteria, carbohydrates, and reduced saliva secretion) and protective factors (antimicrobials, adequate saliva, and remineralizing ions).^125^ The management of WSL occurring during orthodontic treatment should focus on prevention, reducing pathological factors of demineralization, increasing protective factors, and elevating patients’ awareness of maintaining dental hard tissues throughout the entire orthodontic treatment process through oral health education to reduce the occurrence of WSL. For WSL that occurs before or during orthodontic treatment, treatment methods mainly include promoting the remineralization of dental hard tissues, reducing demineralization of dental hard tissues, and restoring aesthetics after demineralization of tooth surfaces.

WSL remineralization treatment promotes the deposition of exogenous calcium and phosphate ions into demineralized crystal voids to increase the mineral content. According to the principle of biomimetic remineralization, polymer membrane modifications are used to induce hydroxyapatite deposition directly on demineralized enamel or dentin surfaces or by utilizing polymer additives such as casein phosphopeptide (CPP), carboxymethyl chitosan, amelogenin-like proteins, polyaspartic acid (PASP), and polyacrylic esters (PAA), simulating noncollagen proteins (NCPs) or nonenamel proteins in the biological mineralization process by stabilizing precursor phases and promoting precursor ion infiltration, mediating the process of transforming amorphous calcium phosphate (ACP) to the crystalline hydroxyapatite mineral phase.^126,127^

For WSL with a Gorelick score of 3 or higher, it is difficult for remineralization treatment to restore enamel defects on the tooth surface. The current treatment options mainly involve selecting minimally invasive aesthetic restorations based on the size of the defect or veneer restorations to address aesthetic concerns. Minimally invasive aesthetic restorations involve abrasing white chalky lesions on the tooth surface with microabrasive paste and restoring tooth morphology with infiltrating resin. Veneers entail uniformly removing surface demineralized discoloured tooth tissue and covering the tooth surface with veneers made of ceramic materials similar in color to healthy tooth tissue using bonding techniques to achieve restoration effects. Although aesthetic restorations can maximally restore tooth surface morphology, irreversible damage is also caused to the tooth structure itself during the removal of diseased tissue. Modern caries management focuses on primary prevention, achieving and maintaining tooth health by optimizing clinical decisions, stopping the progression of initial lesions, preserving tooth structure, and resorting to filling therapy only when necessary.^128^

The biomimetic remineralization method simulating natural mineralization processes provides a promising strategy for the treatment of WSL. And Yuxing Bai et al. used nanostructured resin infiltrant containing calcium phosphate nanoparticles to treat WSL of enamel and achieved satisfactory results.^129^ However, normal enamel and dentin structures are difficult to form, and the remineralized areas on the surfaces of demineralized enamel and dentin, which are generally less than 10 µm thick, are too thin to resist occlusal forces and the mechanical friction of food mastication. Research on the treatment of dental hypoplasia is still in its early stages. The occurrence and development of WSL have become oral health issues that cannot be ignored in orthodontic processes. The development of restoration materials that promote the biomimetic remineralization of WSL and possess excellent mechanical properties holds broad application prospects for WSL treatment.

With the comprehensive arrival of the artificial intelligence era, orthodontics and the field of artificial intelligence are continuously emerging, driving the development and innovation of orthodontics. In the future, it will be possible to utilize AI-based technological methods, in conjunction with existing examination methods for WSL, to personalize the monitoring of teeth during orthodontic processes. This can predict the development and prognosis of WSL at an early stage, alert patients to the risk of WSL occurrence, and further reduce the impact of WSL on orthodontic processes. At the same time, orthodontists need to realize that artificial intelligence only plays a supplementary role in orthodontic processes. Various emerging technologies cannot fully replace the role of orthodontists in preventing and diagnosing WSL. Orthodontists still need to enhance their understanding of WSL, identify early signs of chalky lesions promptly, and intervene when necessary.
